# Characterizations of Anti-Alpha-Fetoprotein-Conjugated Magnetic Nanoparticles Associated with Alpha-Fetoprotein for Biomedical Applications

**DOI:** 10.3390/s17092018

**Published:** 2017-09-03

**Authors:** Shu-Hsien Liao, Han-Sheng Huang, Jen-Jie Chieh, Yu-Kai Su, Yuan-Fu Tong, Kai-Wen Huang

**Affiliations:** 1 Institute of Electro-Optical Science and Technology, National Taiwan Normal University, Taipei 116, Taiwan; hansheng9527@gmail.com (H.-S.H); jjchieh@ntnu.edu.tw (J.-J.C.); a20296111@gmail.com (Y.-K.S.); 60548011s@ntnu.edu.tw (Y.-F.T.); 2 Department of Surgery and Hepatitis Research Center, National Taiwan University Hospital, Taipei 100, Taiwan; 3 Graduate Institute of Clinical Medicine, National Taiwan University, Taipei 100, Taiwan

**Keywords:** magnetic immunoassay, biofunctionalized magnetic nanoparticles, biomarker, alpha-fetoprotein, hepatocellular carcinoma, magnetization enhancement

## Abstract

In this work, we report characterizations of biofunctionalized magnetic nanoparticles (BMNPs) associated with alpha-fetoprotein (AFP) for biomedical applications. The example BMNP in this study is anti-alpha-fetoprotein (anti-AFP) conjugated onto dextran-coated Fe_3_O_4_ labeled as Fe_3_O_4_-anti-AFP, and the target is AFP. We characterize magnetic properties, such as increments of magnetization ΔM_H_ and effective relaxation time Δτ_eff_ in the reaction process. It is found that both ΔM_H_ and Δτ_eff_ are enhanced when the concentration of AFP, Ф_AFP_, increases. The enhancements are due to magnetic interactions among BMNPs in magnetic clusters, which contribute extra M_H_ after the association with M_H_ and in turn enhance τ_eff_. The screening of patients carrying hepatocellular carcinoma (HCC) is verified via ΔM_H_/M_H_. The proposed method can be applied to detect a wide variety of analytes. The scaling characteristics of ΔM_H_/M_H_ show the potential to develop a vibrating sample magnetometer system with low field strength for clinic applications.

## 1. Introduction 

Immunoassays are biochemical tests used to detect or quantify a specific substance, such as analytes in samples of blood or bodily fluid, using immunological reactions. Immunoassay methods include the enzyme-linked immunosorbent assay (ELISA) [1], radioimmunoassay (RIA) [2], real-time polymerase chain reaction (real-time PCR) [3], immunonephelometry [4], etc. Some immunoassays, such as ELISA, require two antigens and separation of the unbound antigens, which can be tedious and time-consuming. On the other hand, magnetic immunoassay (MIA) is a novel type of diagnostic technology using magnetic nanoparticles (MNPs) as labels to replace conventional ELISA, RIA, real-time PCR, etc. MNPs are coated with dextran so that they are encapsulated or glued together with polymers in sizes of nanometers or even micrometers. In immunomagnetic tests, MNPs are first biofunctionalized against antibodies to target antigens. Reagents consisting of biofunctionalized magnetic nanoparticles (BMNPs) are then mixed with samples. Due to the molecular interactions among BMNPs and biomarkers, magnetic clusters are conjugated in the reaction process and their magnetic properties change after the association. The magnetic signal due to the changes of magnetic properties is analyzed in order to determine the unknown amount of biomarkers. Magnetic properties (magnetic relaxation [5,6], remanent magnetization [7], Brownian relaxation [8], saturation magnetization [9], spin-spin relaxation of NMR [10], and alternative-current (AC) susceptibility reduction [11,12,13,14,15], etc.) have been developed recently. Magnetic immunoassays can be carried out simply by mixing reagents and tested samples together and taking physical measurements. Additionally, the background noise of magnetic detection is negligible; hence, high detection sensitivity can be achieved.

Based on the increment of saturation magnetization, ΔM_S_, Chieh et al. [16] recently reported another assay method that used a vibrating sample magnetometer (VSM) to label tumor biomarkers of alpha-fetoprotein (AFP) in clinical studies via the ΔM_S_/M_S_-versus-Ф_AFP_ curve at the saturation field H_S_, where Ф_AFP_ was the concentration of AFP. The authors demonstrated that VSM can be used to screen patients carrying hepatocellular carcinoma (HCC) with sensitivity better than the criterion set in clinics (0.02 μg/mL). It would be interesting to see whether we can screen HCC patients with high detection sensitivity at low magnetic fields (H). Therefore, in this work, we propose a detection method based on the scaling characteristic of the normalized increment of magnetization at low magnetic fields. It is found that M_AFP_ and τ_eff_ are enhanced when Ф_AFP_ increases, where M_AFP_ is the magnetization of the reagent and τ_eff_ is the effective relaxation time. We attribute those results to the molecular interactions among BMNPs in the associated magnetic clusters, which contribute extra magnetization and in turn enhance τ_eff_. The scaling characteristic of (ΔM_AFP_/M_AFP,0_)-versus-Φ_AFP_ curves at low magnetic fields is demonstrated, and the screening of HCC patients via the scaling characteristic is verified in clinical studies.

## 2. Experiments

The MNPs in this study were dextran-coated Fe_3_O_4_ (MF-DEX-0060, MagQu Co., Ltd., New Taipei City, Taiwan) with a mean core diameter of ~35 nm, as detected by x-ray diffraction (D-500, Siemens). The BMNPs were Fe_3_O_4_-anti-AFP (MF-AFP-0060, MagQu Co. Ltd., New Taipei City, Taiwan), and the biotarget was AFP, which is a biomarker for diagnosing HCC. When the AFP level is abnormally high before surgery or other therapy, it is expected to fall to normal levels following the successful removal of all cancer cells. 

In performing the AFP tests, the BMNPs consisting of Fe_3_O_4_-anti-AFP were first mixed with AFP. The changes of magnetic properties after the reaction process were then characterized using a VSM (Model Hystermag, MagQu Co., Taiwan) and AC susceptometer. The data of the normalized increments of magnetization ΔM/M were analyzed for a magnetic immunoassay. The AC susceptibility was measured by a highly balanced AC susceptometer in order to monitor the real-time reaction process. The AC susceptibility χ_ac_(ω) can be expressed as follows:

χ_ac_ = χ’ + iχ’’ (1)

where i = (−1)^1/2^, χ’’/χ’ = tanθ = ωτ_eff_(t), and θ is the phase lag of the time-varying magnetization M(t) with respect to the applied AC magnetic field H(t).

**Figure 1:**
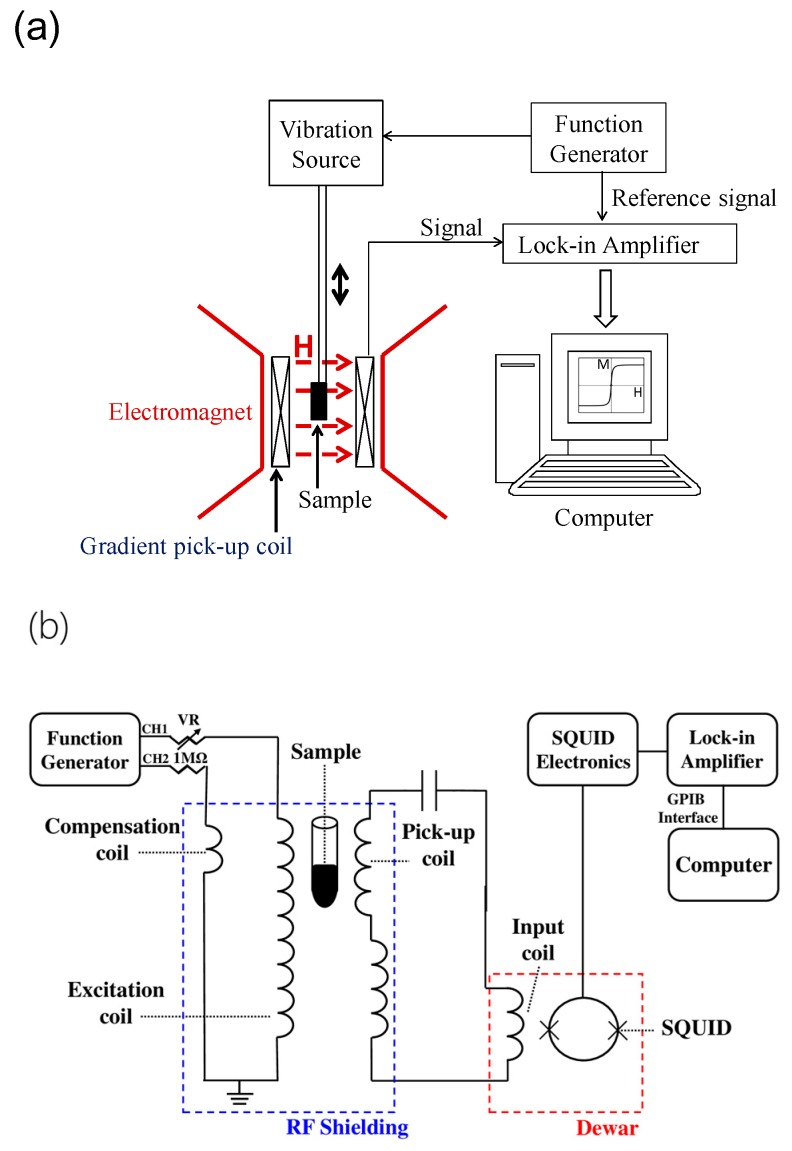
Detection scheme of (**a**) vibrating sample magnetometer; (**b**) high-Tc SQUID-based AC susceptometer.

Figure 1a shows the detection schematic of the VSM used for characterizing M after the BMNPs had conjugated with AFP. In the measurement of M, the sample vibrated with a frequency of ~30 Hz. The magnetic signal was detected with a second-order gradient coil. An electromagnet provided a magnetic field of up to 1.0 Tesla, so that the M–H curves of reagents were characterized. In assaying AFP, a reagent composed of 40 μL Fe_3_O_4_-anti-AFP was mixed with 60 μL AFP. We measured the M–H curves and analyzed the magnetization enhancement (ΔM) at low external fields (H) to establish the relationship between ΔM/M and the concentrations of AFP (Ф_AFP_). Figure 1b shows the high-T_C_ SQUID-based AC susceptometer for characterizing the AC magnetic susceptibility. The excitation frequency is ~16 kHz. The magnetic signal of BMNPs is picked up by a gradient coil that is coupled to a high-T_C_ SQUID via a flux transformer. The detailed design of the pickup coil, gradient coil, and compensation coil in a homemade AC susceptometer that did not use a high-T_c_ SQUID was reported in [17,18].

**Figure 2:**
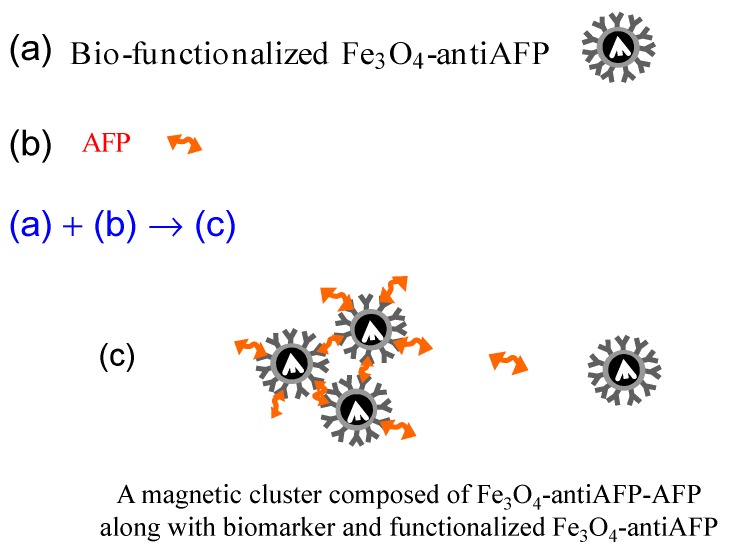
Pictures showing (**a**) biofunctionalized Fe_3_O_4_-anti-AFP; (**b**) AFPs; (**c**) magnetic cluster composed of Fe_3_O_4_-anti-AFP-AFP.

The reagent was composed of anti-AFP-conjugated Fe_3_O_4_ labeled as Fe_3_O_4_-anti-AFP. The bio-target was AFP. Figure 2 depicts Fe_3_O_4_-anti-AFP, AFP, and a magnetic cluster composed of Fe_3_O_4_-anti-AFP-AFP.

## 3. Results and Discussion

This section addresses and discusses the results from the characterization of magnetic properties when biofunctionalized Fe_3_O_4_-anti-AFPs are associated with AFP. Additionally, we present the results from the real-time association of Fe_3_O_4_-anti-AFP with AFP via the time-dependency studies of τ_eff_(t) in the reaction process using the technique of AC susceptibility. We also briefly summarize the findings. Finally, we present the clinical research on screening HCC patients via normalized increments of magnetization and address and discuss advances in sensitive bio-sensing.

**Figure 3:**
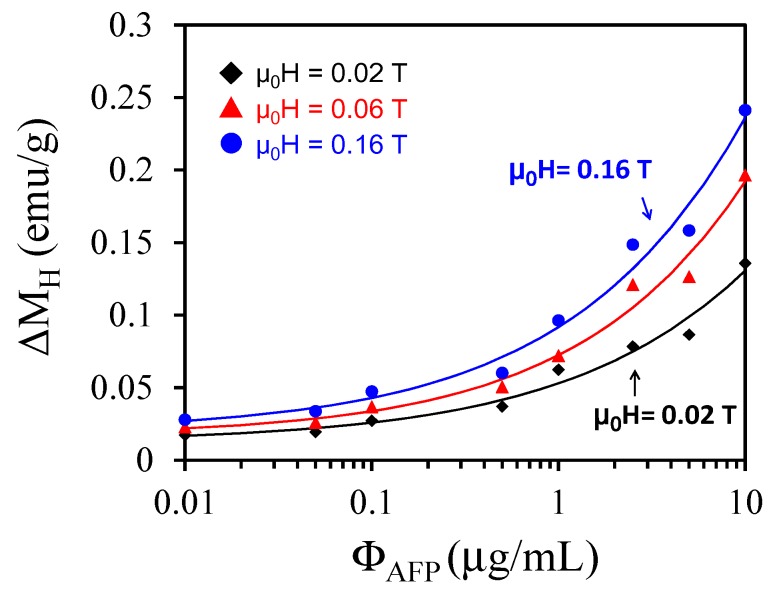
The increments of magnetization ΔM_H_ as a function of Ф_AFP_ at low magnetic fields at μ_0_H = 0.02 T, 0.06 T, 0.16 T.

Figure 3 shows ΔM_H_ as a function of Ф_AFP_ at μ_0_H = 0.02 T, 0.06 T, and 0.16 T and ΔM_H_ = M_H_(Ф_AFP_) − M_H_(Ф_AFP_ = 0). For a fixed magnetic field at μ_0_H = 0.02 T, ΔM_H_ = 0.015 emu/g when Ф_AFP_ = 0.01 μg/mL, and ΔM_H_ increases to ΔM_μ0H = 0.02 T_ = 0.13 emu/g when Ф_AFP_ = 10 μg/mL. For μ_0_H = 0.16 T, ΔM_μ0H = 0.16 T_ = 0.03 emu/g when Ф_AFP_ = 0.01 μg/mL, and ΔM_H_ increases to ΔM_μ0H = 0.16 T_ = 0.23 emu/g when Ф_AFP_ = 10 μg/mL. Hence, we have demonstrated an enhancement of ΔM_H_ when Ф_AFP_ increases at a fixed magnetic field. We attribute those enhancements to the fact that more magnetic clusters are associated and stronger magnetic interactions among BMNPs are present.

**Figure 4:**
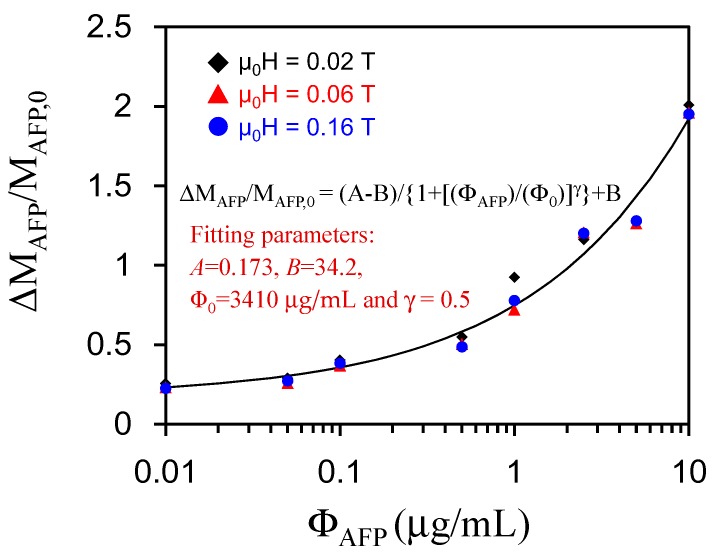
The normalized increment of magnetization ΔM_AFP_/M_AFP,0_ as a function of Ф_AFP_ with data analyzed at μ_0_H = 0.02 T, 0.06 T and 0.16 T.

Figure 4 shows the normalized increment of magnetization, ΔM_AFP_/M_AFP,0_, as a function of Ф_AFP_ at μ_0_H = 0.02 T, 0.06 T, and 0.16 T, where ΔM_AFP_ = M(Ф_AFP_) − M(Ф_AFP_ = 0), M_AFP,0_ = M_H_(Ф_AFP_ = 0). It is found that ΔM_AFP_/M_AFP,0_ as a function of Ф_AFP_ in external magnetic fields can be scaled to a universal logistic function described by the following formula [15]:

ΔM_AFP_/M_AFP,0_ = (A − B)/{1 + [(Ф_AFP_)/(Ф_0_)]^γ^} + B (2)

where A and B are dimensionless quantities and Ф_0_ is dimensionless. The fitting parameters are as follows: A = 0.173, B = 34.2, Φ_0_ = 3410 μg/mL, and γ = 0.5. We have established a relationship between ΔM_AFP_/M_AFP,0_ and Ф_AFP_ with Ф_AFP_ varied from 0.01 μg/mL to 10 μg/mL. Therefore, the unknown amounts of AFP can be determined via a scaling characteristic of the (ΔM_AFP_/M_AFP,0_)-versus-Ф_AFP_ curve, which is versatile and can be applied to assay other biomarkers. In assaying other biomarkers, the relationship between ΔM_biomarker_/M_biomarker,0_ and Ф_biomarker_ is first established and then ΔM_biomarker_/M_biomarker,0_ and the Ф_biomarker_ curve are applied to determine the unknown amount of biomarkers quantitatively.

**Figure 5:**
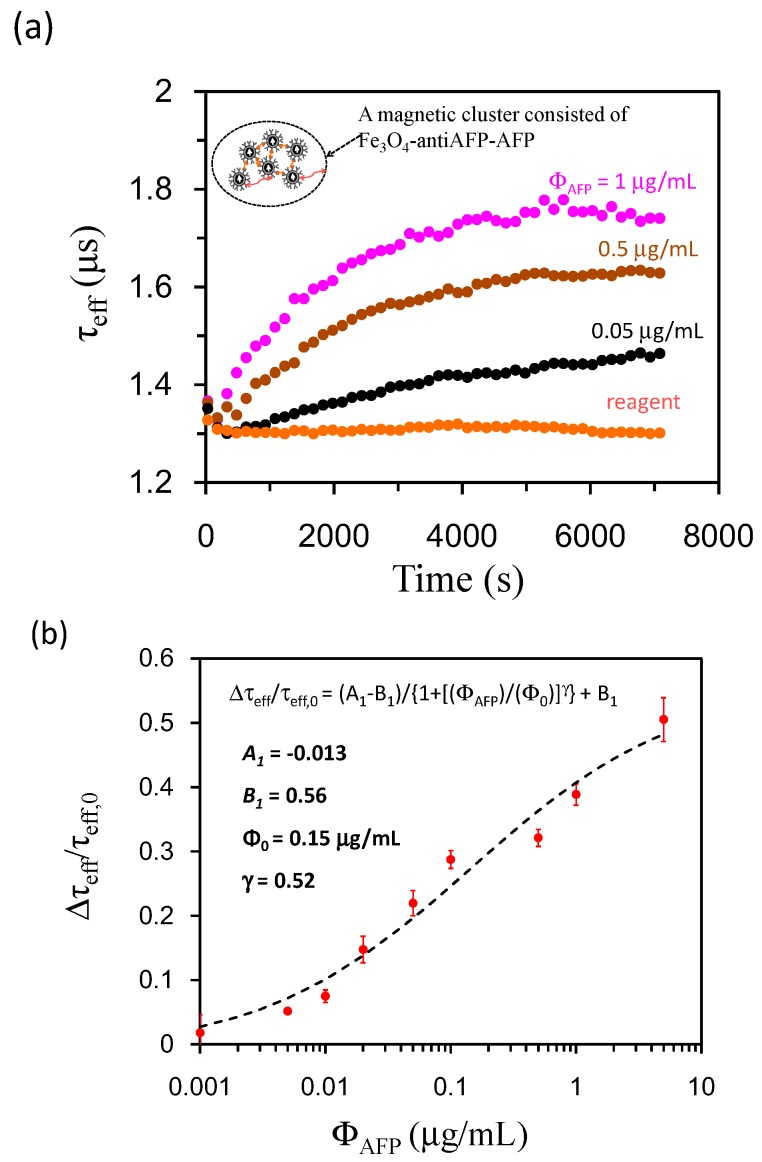
(**a**) τ_eff_ as a function of time, (**b**) Δτ_eff_/τ_eff,0_ as a function of Ф_AFP_ with Ф_AFP_ from Ф_AFP_ = 0.001 μg/mL to Ф_AFP_ = 1 μg/mL.

To observe the real-time association of τ_eff_ when Fe_3_O_4_-anti-AFPs are associated with AFP directly, we characterize the time-dependent τ_eff_ via the following formula: tanθ = ωτ_eff_, where χ’’/χ’ = tanθ and χ’ and χ’’ are the real and imaginary parts of AC susceptibility in Equation (1). Figure 5a shows τ_eff_(t) as a function of time in the reaction process. The reagent shows τ_eff_ = ~1.3 μs, and τ_eff_ is stable to τ_eff_ = 1.3 μs at t = 7200 s. It takes approximately 6000 s for the reagent to complete the association and τ_eff_ is increased to τ_eff_ = ~1.75 μs with Ф_AFP_ = 1 μg/mL. Therefore, a detection time of 7200 s is suggested. The real-time association of Fe_3_O_4_-anti-AFP with AFP is verified. 

The Brownian relaxation time, τ_B_, is a function of the hydrodynamic volume of a magnetic particle, V_H_, the viscosity of the medium, η, the Boltzmann’s constant, k, and the absolute temperature, T, which is expressed as follows [19]:

τ_B_ = 3 V_H_η/kT (3)

In the reaction process, we assume that the viscosity and temperature are constant. The Brownian relaxation time is proportional to the hydrodynamic volume of the magnetic particle. The ratio of the increase in τ_eff_ after the reaction process is 1.35 with an Ф_AFP_ value of 1 μg/mL. The effective diameter of the magnetic cluster is 2.4 times larger than a single magnetic particle when Ф_AFP_ is 1 μg/mL. It presents the formation of magnetic clusters during the reaction process.

Figure 5b shows Δτ_eff_/τ_eff,0_ as a function of Ф_AFP_ with Ф_AFP_ ranging from Ф_AFP_ = 0.001 μg/mL to Ф_AFP_ = 1 μg/mL. The reagent shows τ_eff_ = 1.3 μs, and τ_eff_ is enhanced to τ_eff_ = ~1.75 μs when Ф_AFP_ = 1 μg/mL. The enhancement of τ_eff_ is due to the presence of magnetic clusters in the reaction process. The magnetic interaction among BMNPs enhances M, which in turn increases τ_eff_. The (Δτ_eff_/τ_eff,0_)-versus-Ф_AFP_ curve follows the characteristic curve [15]:

Δτ_eff_/τ_eff,0_ = (A_1_ − B_1_)/{1+[(Ф_AFP_)/(Ф_0_)]^γ^} + B_1_, (4)

where Δτ_eff_ = τ_eff_(7200 s) − τ_eff_(t = 0) and τ_eff,0_ = τ_eff_(t = 0). The curve is fitted to the following parameters: A_1_ = −0.013 μs, B_1_ = 0.56 μs, Φ_0_ = 0.15 μg/mL, and γ = 0.52. Equation (4) reveals the concentration dependency of the characteristic of Δτ_eff_/τ_eff,0_ after the BMNPs have completed the association with AFP. The (Δτ_eff_/τ_eff,0_)-versus-Φ_AFP_ curve shown in Figure 5b can be applied to screening patients carrying HCC. Normalized Δτ_eff_/τ_eff,0_ is analyzed instead of Δτ_eff_ for a magnetic immunoassay, because this enables us to eliminate minor differences in magnetic signals due to minor differences in sample amounts used from run to run, which will enhance the detection sensitivity. 

Detection sensitivity can be defined by the noise level with standard deviations for the detected signal at low concentrations [20]. In this study, the detection sensitivity levels are 0.0024 μg/mL and 0.0177 μg/mL, as determined by measuring Δτ_eff_/τ_eff,0_ and ΔM_AFP_/M_AFP,0_ respectively. The reference criterion of the AFP serum level for HCC is 0.02 μg/mL. The sensitivity of both methods reaches the criteria for a clinical AFP assay. The feasibility of AFP is demonstrated by measuring Δτ_eff_/τ_eff,0_ and ΔM_AFP_/M_AFP,0_.

In this study, we characterized magnetic properties when BMNPs are associated with AFPs for biomedical applications. The findings in the characterization of magnetic properties are briefly summarized as follows. First, M and τ_eff_ are enhanced when reagents composed of BMNPs are conjugated with AFP in the reaction process. The magnetic interactions among BMNPs in magnetic clusters enhance M, which in turn increases τ_eff_. Second, the real-time association of BMNPs with AFP was demonstrated in the time-dependent τ_eff,_. Third, bio-detection based on the (Δτ_eff_/τ_eff,0_)-versus-Φ_biomarkers_ curve provided a sensitive methodology for assaying unknown amounts of AFP, and BMNPs could be applied to assay large molecules such as AFP as well as small molecules such as C-reactive protein(CRP) [21]. Finally, the proposed detection methodology based on the (Δτ_eff_/τ_eff,0_)-versus-Φ_biomarkers_ curve was versatile, and the (ΔM_AFP_/M_AFP,0_)-versus-Ф_AFP_ curves shown in Figure 4 were scaled to a characteristic function described by Equation (2). The results confirm that both changes in ΔM_AFP_/M_AFP,0_ and Δτ_eff_/τ_eff,0_ are caused by the formation of magnetic clusters and can be applied to sense a wide variety of biomarkers.

**Figure 6:**
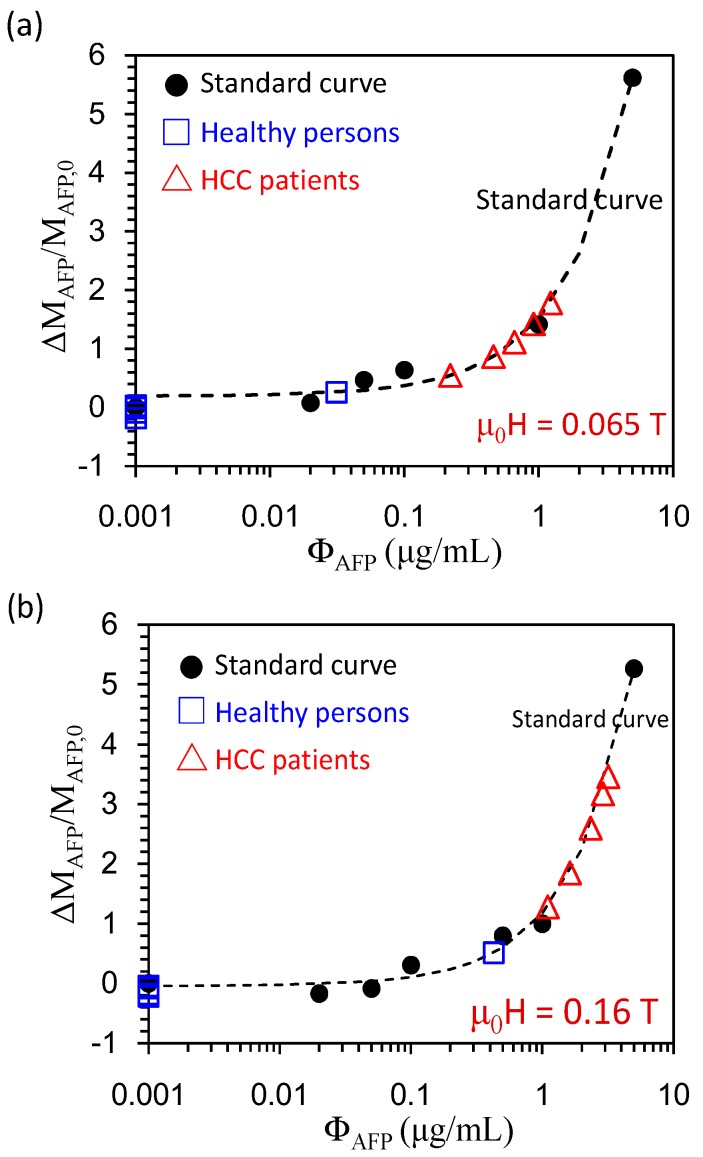
The normalized increment of magnetization ΔM_AFP_/M_AFP,0_ as a function of Ф_AFP_ with data analyzed at μ_0_H = 0.065 T. On the standard curve, AFP levels for healthy persons and HCC patients are shown.

The sensitivity levels of Δτ_eff_/τ_eff,0_ and ΔM_AFP_/M_AFP,0_ reach the criteria for a clinical AFP assay. The cost of a high-T_C_ SQUID-based AC susceptometer is much higher than that of a VSM with a low-strength magnet. The low-strength VSM has high potential for commercial and clinical applications. Therefore, the screening of HCC patients can be addressed by measuring ΔM_H_/M_H,0_. Since the data shown in Figure 4 are scaled to a characteristic function described by Equation (2), it would be interesting to verify whether we can also obtain high detection sensitivity at low magnetic fields via Equation (2). Hence, we can apply Equation (2) at a low magnetic field, say μ_0_H = 0.065 T, to analyze AFP levels in clinical studies. To verify this, we show in Figure 6a (ΔM_AFP_/M_AFP,0_)-versus-Ф_AFP_ with data analyzed at μ_0_H = 0.065 T, where ΔM_AFP_ = M_H_(Ф_AFP_) − M_H_(Ф_AFP_ = 0) and M_AFP,0_ = M_H_(Ф_AFP_ = 0). The background magnetic signal of serum from healthy persons in ΔM_AFP_/M_AFP,0_ is deducted in the data analysis. To screen patients carrying HCC and healthy persons, we mixed 40 μL 0.1 emu/g of reagent with 60 μL of serum. The data for establishing the standard curve are marked with a solid dot (•). AFP levels in serum for HCC patients are marked with an open triangle (**Δ**), while AFP levels for healthy persons are marked with an open square (**□**). The reference criterion of the AFP serum level for HCC is 0.02 μg/mL. We found that the average AFP levels for patients carrying HCC were higher than ~0.2 μg/mL, which is significantly higher than the criterion set in clinics (0.02 μg/mL). The average AFP levels for healthy persons were below ~0.02 μg/mL, except for one healthy person who showed a false positive (AFP level = ~0.03 μg/mL). Figure 6b shows ΔM_AFP_/M_AFP,0_ as a function of Ф_AFP_ with data analyzed at 0.16 T. HCC patients showed AFP levels higher than the clinical criterion. Healthy persons showed AFP levels of 0.001 μg/mL, except for one healthy person with a higher AFP level of ~0.4 μg/mL. The estimated values of Ф_AFP_ were different between μ_0_H = 0.065 T and 0.16 T. It was probably due to the magnetic clustering effect that induces background magnetic noises. Besides, the ΔM_AFP/_ΔM_AFP,0_ of serum tested at 0.16 T is higher than that at 0.065 T. It leads that the estimated AFP concentration at 0.16 T is higher than that at 0.065 T. The reason may be due to the larger background magnetization of serum than that of the AFP solution. The reference magnetization, M(Ф_AFP_ = 0), in the clinical test may be considered by using the averaging magnetization of healthy persons to reduce the effect in the clinical test. Thus, the feasibility of screening HCC patients by assaying AFP levels in serum was verified.

The AFP level in serum was recently determined via the ΔM_S_-versus-Ф_AFP_ curve at the saturation field μ_0_H_S_ = ~0.4 T [16], where ΔM_S_ is the increment of the saturated magnetization. A clear demarcation between the normal group and the HCC group was verified in the test results, which indicates the feasibility of using ΔM_S_-versus-Ф_AFP_ at the saturation field as the primary analysis factor for identifying the AFP risk level in patients. In this work, the screening of HCC patients was fulfilled at low magnetic fields, which makes the detection platform simple for biomedical application users.

## 4. Conclusions

In summary, we performed measurements of magnetization (M–H curves) and AC susceptibility when reagents consisting of Fe_3_O_4_-anti-AFP were conjugated with AFP. The scaling characteristic of (ΔM_AFP_/M_AFP,0_)-versus-Φ_AFP_ curves at low magnetic fields was demonstrated, and bio-sensing using BMNPs via increments of magnetization was proposed. We showed that BMNPs can be applied to assay large as well as small molecules. The screening of HCC patients via the scaling characteristic was verified in clinical studies. The detection mechanism based on the scaling characteristic showed potential to develop a compact VSM with a low magnetic field for biomedical applications.
